# Long‐term analysis of adalimumab in Japanese patients with moderate to severe hidradenitis suppurativa: Open‐label phase 3 results

**DOI:** 10.1111/1346-8138.15605

**Published:** 2020-10-07

**Authors:** Akimichi Morita, Hidetoshi Takahashi, Kentaro Ozawa, Shinichi Imafuku, Nakama Takekuni, Kenzo Takahashi, Takashi Matsuyama, Yukari Okubo, Yiwei Zhao, Susumu Kitamura, Keiko Takei, Masayuki Yokoyama, Nobukazu Hayashi, Tadashi Terui

**Affiliations:** ^1^ Nagoya City University Graduate School of Medical Sciences Nagoya Japan; ^2^ Takagi Dermatological Clinic Hokkaido Japan; ^3^ National Hospital Organization Osaka National Hospital Osaka Japan; ^4^ Fukuoka University Fukuoka Japan; ^5^ Kurume University School of Medicine Fukuoka Japan; ^6^ University of Ryukyus Okinawa Japan; ^7^ Tokai University Tokyo Japan; ^8^ Tokyo Medical University Tokyo Japan; ^9^ AbbVie GK Tokyo Japan; ^10^ Toranomon Hospital Tokyo Japan; ^11^ Nihon University School of Medicine Tokyo Japan

**Keywords:** adalimumab, hidradenitis suppurativa, Japan, quality of life, tumor necrosis factor‐α inhibitor

## Abstract

This phase 3, multicenter, open‐label single‐arm study evaluated adalimumab (ADA) in Japanese patients with moderate to severe hidradenitis suppurativa (HS). Fifteen patients received ADA 160 mg s.c. at week 0, 80 mg at week 2 and 40 mg at week 4 and every week thereafter. At any time after week 52, patients were given the option to receive 80 mg ADA every other week or remain on 40 mg every week. The primary end‐point (achievement of HS Clinical Response [HiSCR] at week 24) and results up to week 24 were published previously. Secondary end‐points included total abscess and inflammatory nodule (AN) count, 30% or more and 1 unit or more reduction in Patient’s Global Assessment of Skin Pain Numeric Rating Scale (NRS30), modified Sartorius score and quality of life (QoL). After 12 weeks of ADA treatment, the achievement rate in HiSCR was 86.7%; HiSCR achievement rate was sustained through week 52 at 66.7%. Improvements at week 12 were also seen in the proportion of patients achieving an AN count of 0–2; NRS30 response rate among the nine patients with a baseline NRS of 3 or more; mean decrease in modified Sartorius score (61.4); and QoL as assessed by Dermatology Life Quality Index and Treatment Satisfaction Questionnaire; these improvements were maintained through 52 weeks. Similar efficacy was observed when patients switched dosing from ADA 40 mg every week to ADA 80 mg every other week. There were no new safety findings with ADA 40 mg weekly dosing during the study, and no differences in safety were found between patients who switched to 80 mg ADA every other week and patients who remained on 40 mg every week. The results of this study indicate that long‐term ADA treatment is effective and well tolerated in Japanese patients with moderate to severe HS.

## INTRODUCTION

Hidradenitis suppurativa (HS) is a difficult to treat, chronic, painful skin disease characterized by deep‐seated inflammatory nodules and abscesses, fistula formation and subsequent scarring, which negatively impacts patients’ quality of life (QoL).^1^, ^2^, ^3^, ^4^ Prevalence of HS in Eastern and Western countries varies. In Eastern countries, HS is 2–3‐fold more prevalent in men than in women, with approximately two‐thirds of patients having Hurley stage II or III disease and the buttocks, axillae and groin areas being the most commonly affected anatomical locations.^5^, ^6^ In contrast, in Western countries, the prevalence of HS is 2–3‐fold higher for women than men, with more than 95% having Hurley stage I or II disease and the most commonly affected areas being the groin and axillae.^7^, ^8^, ^9^ Because differences in patient background, prevalence and incidence have been noted between Western patients and Japanese patients with HS, further epidemiological studies are warranted in Japan.^5^, ^6^

The clinical course of HS varies over the patient’s lifetime. Important patient‐outcome measures include flares that involve odorous, pus‐filled skin lesions that worsen and may burst at unpredictable times.^10^ The long‐term impact of HS on patient QoL goes beyond the associated chronic pain that can sometimes be intense and includes increases in health‐care‐related costs.^11^ HS also negatively effects patients’ daily activities and is associated with several comorbidities, including mental health disorders and cellulitis; therefore, early diagnosis and treatment are clinically important.^4^, ^10^, ^11^, ^12^ Adalimumab (ADA; Humira^®^ [AbbVie, North Chicago, IL, USA]) is a monoclonal antibody against tumor necrosis factor (TNF)‐α, and is the only medication approved in Japan (at a dose of 40 mg every week), the USA and the EU for the treatment of HS.^13^, ^14^, ^15^

In Japan, ADA has been approved to treat rheumatoid arthritis, plaque psoriasis, psoriatic arthritis, Crohn’s disease, ankylosing spondylitis, HS, juvenile idiopathic arthritis, ulcerative colitis, intestinal Behçet’s disease, non‐infectious intermediate uveitis, posterior uveitis or panuveitis and generalized pustular psoriasis. If the 80 mg ADA every other week regimen is approved for HS, as it has been for other indications in Japan (including rheumatoid arthritis, plaque psoriasis, psoriatic arthritis, pustular psoriasis, ankylosing spondylitis and Crohn’s disease), patients could benefit from the more convenient, lower frequency dosing option. Results from the 24‐week interim analysis of this single‐arm, open‐label phase 3 study investigating the safety, efficacy and tolerability of weekly ADA treatment in Japanese patients with moderate to severe HS were previously reported.^16^ Herein, we report the long‐term efficacy, safety and impact on QoL of ADA in Japanese patients with moderate to severe HS, including patients who switched to receive the ADA 80 mg every other week dose after 52 weeks of treatment.

## METHODS

### Patients

Men and non‐pregnant women aged 18 years or older were eligible to enroll in the study if they were diagnosed with HS, had stable disease for 2 months or more at baseline, had lesions in two or more anatomical areas (one of which was judged Hurley stage II or III) and had a total abscess and inflammatory nodule (AN) count of three or more at baseline. Patients were excluded from the study if they had prior ADA treatment or were taking other anti‐TNF therapy, participated in another ADA clinical trial, used topical HS therapies or used concomitant oral analgesics (including opioids) for HS‐related pain within 14 days of the baseline visit. Patients were also excluded if they had received antibiotics (other than permitted oral antibiotics) or systemic non‐biologics for HS for 28 days or less before the baseline visit. Female patients of child‐bearing potential had to have negative serum pregnancy test results at screening and negative urine pregnancy test results on the first day of the study. The full list of inclusion and exclusion criteria was published previously.^16^

### Study design and treatment

**Figure 1:**
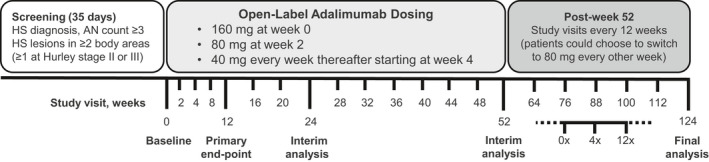
Study design: 124‐week analysis of efficacy, impact on quality of life and safety of adalimumab (ADA) in Japanese patients with moderate to severe hidradenitis suppurativa (HS). Patients who switched to the 80 mg every other week dosing regimen had visits at week 0x, week 4x and week 12x, and then visits every 12 weeks until the study end. AN, total abscess and inflammatory nodule. Adapted from Morita *et al.* ^16^

This phase 3, multicenter, open‐label single‐arm study in Japanese patients with moderate to severe HS evaluated the long‐term efficacy, impact on QoL and safety of ADA therapy (ClinicalTrials.gov: NCT02904902). After the 35‐day screening period and open‐label treatment period, all patients received s.c. injections of ADA 160 mg at week 0 and 80 mg at week 2, followed by 40 mg every week starting at week 4 through 52 weeks (Fig. 1). At any time after week 52, all remaining patients were given the option to switch to 80 mg every other week or continue 40 mg weekly. Patients who consented to receive ADA 80 mg every other week received open‐label s.c. injections starting at week 0x (x indicating the 80 mg every other week period) until the end of this study. These patients had visits at week 0x, week 4x and week 12x, and then visits every 12 weeks until the study end. Patients who switched to 80 mg every other week could return to 40 mg weekly, if it was in their best medical interest as determined by the investigator. Patients received a follow‐up telephone call 70 days after the last study drug dose.

### Assessments

#### Efficacy

The primary efficacy end‐point was the proportion of patients achieving HS Clinical Response (HiSCR),^17^ defined as 50% or more reduction from baseline in AN count with no increase in abscess or draining fistula count at week 12; the proportion of patients achieving HiSCR was also assessed at weeks 2, 4 and every 4 weeks thereafter until week 52. Three secondary end‐points assessed clinical response. These included the proportion of patients achieving an AN count of 0–2; the proportion of patients achieving at their worst 30% or more reduction and 1 unit or more reduction from baseline in the Patient’s Global Assessment of Skin Pain Numeric Rating Scale (NRS30);^2^ and the mean change in modified Sartorius score.^18^ The proportion of patients achieving an AN count of 0–2 and the mean change in modified Sartorius scores were assessed at baseline, weeks 2 and 4 and every 4 weeks until week 52. The NRS30 response rate was assessed among patients with baseline NRS of 3 or more at weeks 2, 4, 8, 12, 24, 36 and 52. NRS30 response was based on patients’ 24‐h recall of the worst skin pain due to HS using an 11‐point scale.

#### Safety

Safety and tolerability assessments included monitoring vital signs, performing standard clinical laboratory tests (hematology, blood chemistry and urinalysis) and recording treatment‐emergent adverse events (AE). The frequency, intensity and relationship of treatment‐emergent AE were recorded at the first study drug dose and up to 70 days after the last dose.

#### QoL

Quality of life was assessed by mean change from baseline using the Dermatology Life Quality Index (DLQI) measured at baseline and at weeks 4, 12, 24, 36, 52, 0x and 12x; the Treatment Satisfaction Questionnaire–Medication (TSQM) given at baseline and at weeks 12, 24, 36, 52, 0x and 12x; the Hidradenitis Suppurativa Quality of Life (HS QoL) questionnaire given at baseline and weeks 2, 4, 8, 12, 24, 36, 52, 0x and 12x; and the 5‐dimensional EuroQol EQ‐5D^19^ that assesses mobility, self‐care, usual activities, pain/discomfort and anxiety/depression, which was given at baseline, weeks 12, 52, 0x and 12x.

### Statistical analysis

Assuming a response rate with no medication at week 12 of 25% based on the clinical response rate for patients in the placebo arm (26.0–27.6%) versus the ADA treatment arm (41.8–58.9%) at week 12 in the PIONEER studies,^2^ a sample size of 13 patients was calculated as necessary to adequately power the study at 80.1% to detect a clinical response (defined as a 35% difference in the primary end‐point) using a one‐sample χ^2^‐test (one‐sided, α = 0.025). Efficacy outcomes were analyzed using the full analysis set population, defined as all patients who received one dose or more of study drug and had one or more post‐treatment efficacy assessments. The safety population was defined as all patients who received one dose or more of the study drug. For safety analysis, all AE with an onset date from the first dose through 70 days after the last dose of ADA 40 mg every week were included. The only exceptions were data collected after a patient switched to ADA 80 mg every other week, which were excluded from the safety analysis set and summarized in the 80 mg every other week set. Statistical analysis (two‐tailed, α = 0.05) was performed using SAS statistical software (SAS Institute, Cary, NC, USA). The proportion of patients achieving HiSCR, AN count of 0–2 and NRS30 response rate at each assessment time point were reported using a 95% confidence interval. Missing data were handled using the non‐responder imputation method for all efficacy variables, except for the modified Sartorius score (where missing data were handled by last observation carried forward (LOCF) for the entire study duration, including those patients who switched to 80 mg every other week) through week 52, and by cases as observed after week 52 until the end of the study for patients who remained on the 40 mg every week dosing regimen. Missing data for patients who switched to 80 mg every other week were also handled using the non‐responder imputation method. For all QoL assessments, missing data were handled using the LOCF method before week 52 and by observed cases after week 52.

## RESULTS

### Patients

A total of 15 patients were enrolled across eight study sites in Japan. Patients were predominantly male (87%, *n* = 13) with a mean age of 42 years (range, 26–52) and median body mass index of 26.5 kg/m^2^ (range, 18.1–40.6). Most patients were also current smokers (80.0%) and alcohol users (86.7%). All patients had Hurley stage II or III disease at baseline with a median disease duration of 11.7 years and nine patients (60%) had received prior systemic treatment for HS (Table 1). One patient was discontinued after withdrawing consent because of an AE of cellulitis before week 24. Six patients chose to switch to receiving 80 mg ADA every other week starting at various weeks after week 52 (one at week 76, two at week 88 and three at week 112) until the end of the study, and none returned to the dosing regimen of 40 mg ADA every week. Patients’ characteristics were generally well balanced between dosing regimens, except that more patients who switched to 80 mg every other week had Hurley stage II disease (83%, *n* = 5 vs 38%, *n* = 3), numerically more patients had non‐draining fistulas and inflammatory nodules and higher mean modified Sartorius score (157 vs 126) than did those patients in the 40 mg every week safety analysis set.

**Table 1:** Baseline demographics and disease characteristics

Characteristic	ADA 40 mg every week up to week 52 (*n* = 15)	ADA 40 mg every week after week 52 (*n* = 8)	ADA 80 mg every other week after week 52 (*n* = 6)
Sex, *n* (%)			
Male	13 (87)	7 (88)	5 (83)
Female	2 (13)	1 (13)	1 (17)
Age, years	42.1 (6.94)	42.1 (7.02)	40.7 (7.15)
Median age (range), years	44 (26–52)	44 (26–48)	40 (31–52)
Median BMI (range), kg/m^2^	26.5 (18.1–50.6)	24.4 (18.1–40.6)	27.2 (21.8–33.7)
Hurley stage, *n* (%)			
II	9 (60)	3 (38)	5 (83)
III	6 (40)	5 (63)	1 (17)
HS duration, years	14.1 (10.58)	16.2 (12.67)	13.3 (7.02)
C‐reactive protein, mg/L	8.3 (11.81)	12.1 (15.29)	3.6 (3.66)
Prior HS systemic medication, *n* (%)	9 (60)	5 (63)	3 (50)
Prior HS surgery, *n* (%)	6 (40)	3 (38)	3 (50)
HS lesion count			
Abscess	2.8 (3.43)	3.5 (3.96)	2.3 (2.88)
Draining fistula	3.0 (3.87)	3.0 (2.33)	3.5 (5.68)
Non‐draining fistula	7.5 (6.42)	6.4 (5.97)	10.2 (6.62)
Inflammatory nodule	7.5 (6.08)	4.4 (2.77)	9.3 (6.06)
Hypertrophic scar	8.1 (9.39)	9.1 (11.31)	6.3 (7.87)
Modified Sartorius score	137.3 (59.21)	125.9 (53.72)	157.0 (70.44)
NRS of skin pain due to HS at worst among patients with baseline NRS of ≥3	4.6 (0.60)	4.4 (0.55)	5.2 (0.51)

Data are presented as mean (standard deviation), unless otherwise noted. ADA, adalimumab; BMI, body mass index; DLQI, Dermatology Life Quality Index; HS, hidradenitis suppurativa; NRS, numeric rating scale.

### Efficacy

**Figure 2:**
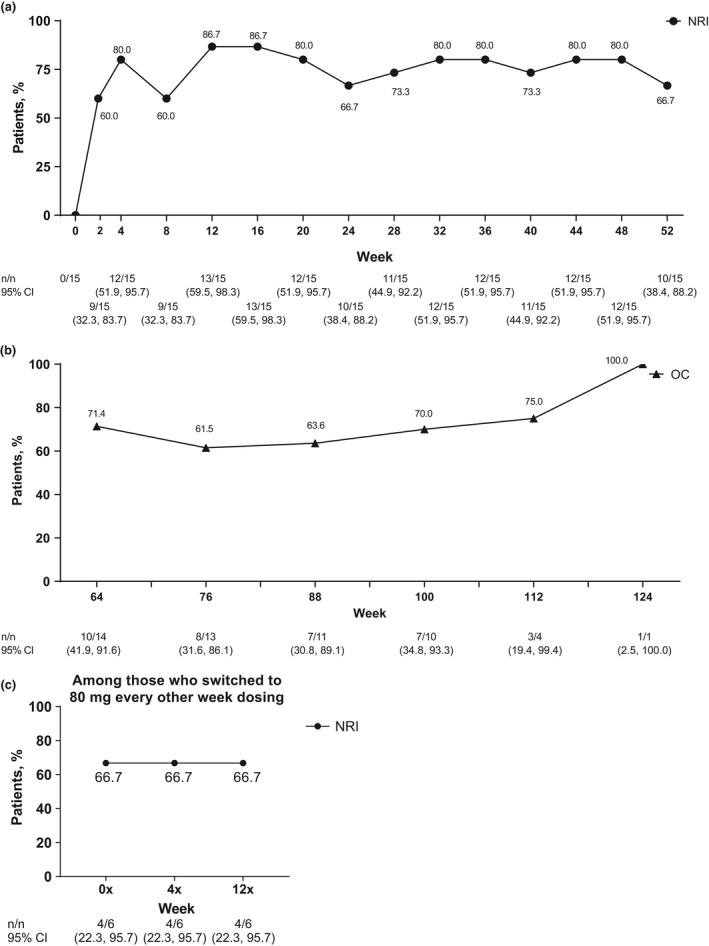
Achievement of Hidradenitis Suppurativa Clinical Response (HiSCR) among patients (a) treated with weekly adalimumab (ADA) 40 mg through 52 weeks (b) treated with weekly ADA 40 mg from 64 through 124 weeks and (c) who switched to ADA 80 mg every other week. Missing data were handled using (a,c) the non‐responder imputation (NRI) method or (b) data are shown as observed cases (OC). CI, confidence interval.

**Figure 3:**
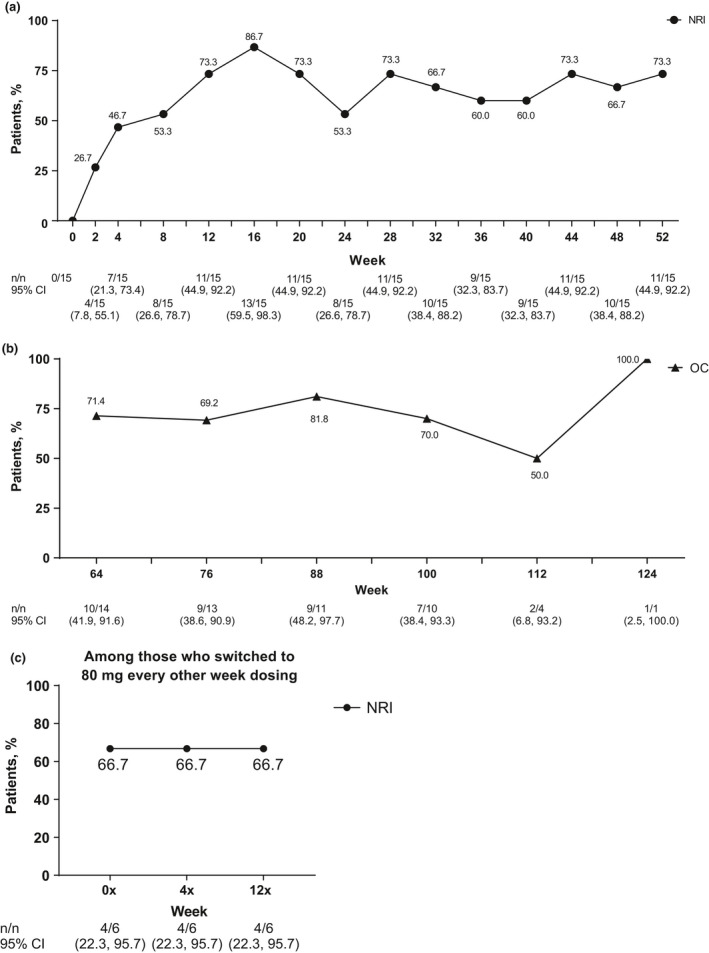
Achievement of total abscess and inflammatory nodule count of 0–2 at each visit among patients (a) treated with weekly adalimumab (ADA) 40 mg through 52 weeks, (b) treated with weekly ADA 40 mg from 64 through 124 weeks and (c) who switched to ADA 80 mg every other week. Missing data were handled using (a,c) the non‐responder imputation (NRI) method or (b) data are shown as observed cases (OC). CI, confidence interval.

**Figure 4:**
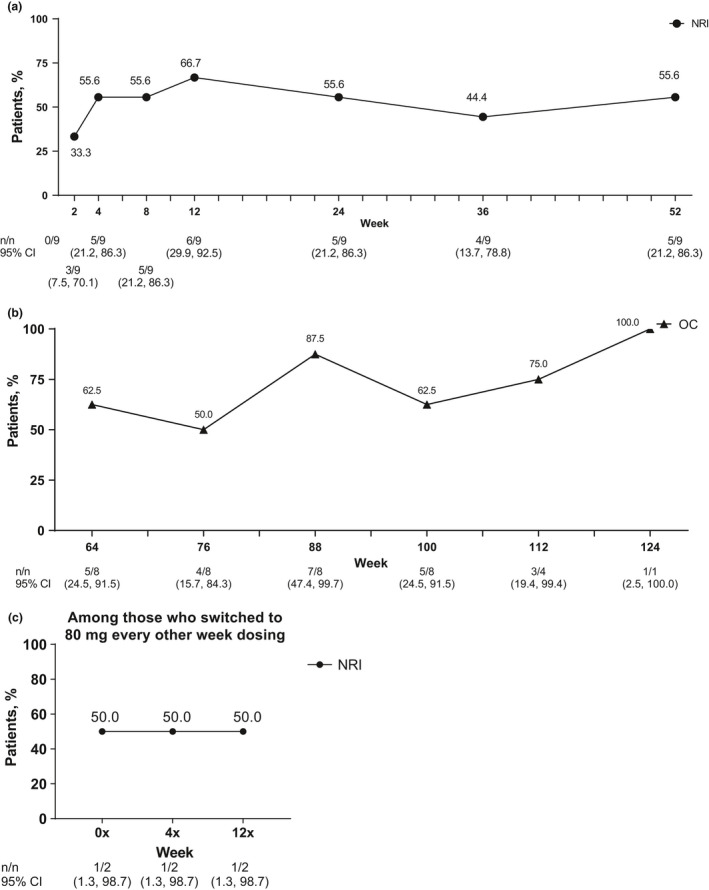
NRS30 response rate among patients with baseline Numeric Rating Scale (NRS) of 3 or more (at worst) among patients (a) treated with weekly adalimumab (ADA) 40 mg through 52 weeks, (b) treated with weekly ADA 40 mg from 64 through 124 weeks and (c) who switched to ADA 80 mg every other week. Missing data were handled using (a,c) the non‐responder imputation (NRI) method or (b) data are shown as observed cases (OC). NRS30, 30% or more reduction and 1 unit or more reduction from baseline in Patient’s Global Assessment for Skin Pain NRS.

**Figure 5:**
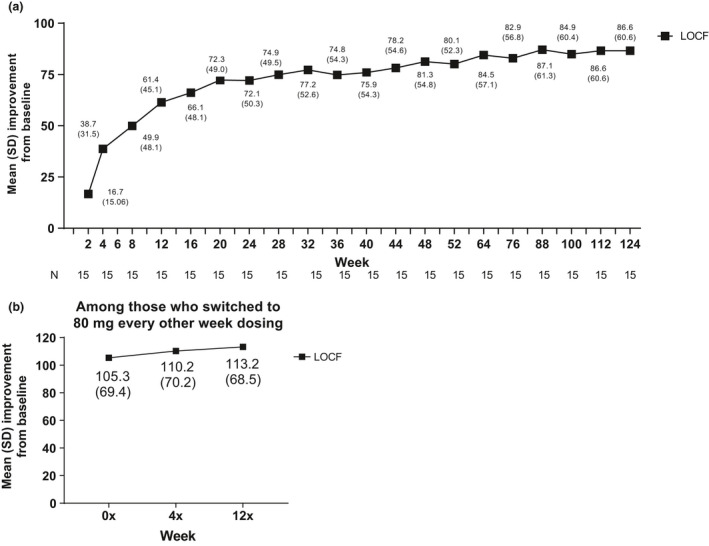
Mean (standard deviation [SD]) improvement in modified Sartorius scores among patients (a) treated with weekly adalimumab (ADA) 40 mg through week 124 and (b) who switched to ADA 80 mg every other week. (a,b) Missing data were handled using the last observation carried forward (LOCF) method.

Most patients met primary efficacy end‐point outcome of achievement of HiSCR at week 12 (86.7%, *n* = 13). Patients initially achieved HiSCR starting at week 2 (60.0%, *n* = 9), and an HiSCR response rate of more than 60.0% was maintained every 4 weeks after through week 52, with an 80.0% or more HiSCR response rate at eight of the 13 time points (Fig. 2a). The HiSCR response rate was also generally maintained through 124 weeks (Fig. 2b). Four of six patients who switched to 80 mg every other week maintained HiSCR response from week 4x through the end of the study (Fig. 2c). Improvements were also seen in the secondary efficacy end‐points of the proportion of patients achieving an AN count of 0–2, the NRS30 response rate and the mean change in modified Sartorius score. Most (73.3%, *n* = 11) patients achieved an AN count of 0–2 at week 12, and this was generally sustained through week 52 (Fig. 3a). The proportion of patients who achieved an AN count of 0–2 was also generally maintained from week 64 to 124 (Fig. 3b). Four of six patients who switched to 80 mg every other week maintained achievement of an AN count of 0–2 from week 4x through the end of the study (Fig. 3c). Among the nine patients with baseline skin pain of NRS of 3 or more, the NRS30 response rate was 33.3% at week 2, 66.7% at week 12 and 55.6% at week 52 (Fig. 4a), and the response was generally maintained for 124 weeks (Fig. 4b). One of two patients with baseline skin pain of NRS of 3 or more who switched to 80 mg every other week achieved NRS30 response at week 0x through the end of the study (Fig 4c). Patients’ mean modified Sartorius score at week 12 decreased by 61.4 from baseline, and decreased by 80.1 at week 52 and 86.6 by week 124 (Fig. 5a). Patients who switched to 80 mg every other week showed a 113.2 mean decrease in modified Sartorius score at week 12x (Fig. 5b).

### Safety

All 15 patients experienced at least one AE of a total of 91 AE reported, and most (87%) were mild or moderate in severity (Table 2). Sixteen AE were reported for more than one patient, with the most frequent AE being influenza and nasopharyngitis. Patients who switched to ADA 80 mg every other week experienced four AE with no events occurring in more than one patient. A total of seven patients taking ADA 40 mg every week experienced 14 AE that were considered to be possibly related to ADA.

**Table 2:** Treatment‐emergent adverse events in Japanese patients treated with adalimumab

Parameter	Patients, *n* (%), 40 mg every week (*n* = 15)		Patients, *n* (%), 80 mg every other week (*n* = 6)	
	Overall	Possibly related to study drug	Overall	Possibly related to study drug
Any AE	15 (100)	7 (47)	4 (67)	1 (17)
Mild	5 (33)	4 (27)	2 (33)	0
Moderate	8 (53)	3 (20)	1 (17)	0
Severe	2 (13)	0	1 (17)	1 (17)
Any serious AE^†^	4 (27)	2 (13)	1 (17)	1 (17)
Cellulitis	2 (13)	2 (13)	0	0
Subcutaneous abscess	1 (7)	0	0	0
Deep vein thrombosis	1 (7)	0	0	0
Uterine leiomyoma	1 (7)	0	0	0
Interstitial lung disease	0	0	1 (17)	1 (17)
AE leading to discontinuation	1 (7)	1 (7)	0	0
Cellulitis	1 (7)	1 (7)	0	0
Preferred term (occurring in more than one patient)				
Influenza	5 (33)		4 (67)	
Nasopharyngitis	5 (33)		1 (17)	
Periodontitis	4 (27)		1 (17)	
Constipation	2 (13)		0	
Dental caries	2 (13)		0	
Diarrhea	2 (13)		0	
Toothache	2 (13)		0	
Pyrexia	2 (13)		0	
Swelling	2 (13)		0	
Cellulitis	2 (13)		0	
Pulpitis dental	2 (13)		0	
Subcutaneous abscess	2 (13)		0	
Back pain	2 (13)		0	
Headache	2 (13)		0	
Eczema	2 (13)		0	
Pruritis	2 (13)		0	

^†^ Patients could have more than one serious adverse event (AE).

Five patients experienced a total of six serious AE, including serious cellulitis (two events), subcutaneous abscess, deep vein thrombosis, uterine leiomyoma and interstitial lung disease. Of these, three were considered to have a reasonable likelihood of being related to the study drug, including the two AE of cellulitis in the 40 mg every week group and one of interstitial lung disease in a patient who switched to the 80 mg every other week dosing regimen. One patient who received ADA every week discontinued from the study after withdrawing consent because of moderate cellulitis that required hospitalization. This patient, who had a history of cellulitis, was injured in the right leg in an area that partly overlapped with the HS‐affected region 1 day prior to developing symptoms on day 106. The study drug was discontinued (last dose given on day 99). Symptoms improved with antibiotic treatment; however, pain and warmth persisted. During hospitalization the patient experienced breathing difficulty accompanied by fever; a chest X ray revealed mild heart enlargement, but echocardiography showed no significant findings. On day 117, the patient was discharged from the hospital. The event was considered ongoing at the end of study. On day 322, the patient reported that fever and warmth of right leg had resolved but mild swelling persisted; based on this, the investigator considered the event recovering. The second patient developed cellulitis on the buttock in the HS‐affected and surrounding regions on day 112. The study drug was interrupted (last dose given on day 134) while the patient was treated with antibiotics. On day 171, the patient was hospitalized and treated with skin incision. On day 172, the patient was discharged from the hospital. The study drug was resumed on day 189 without recurrence of the event. On day 350, the event was considered resolved. Both events were considered ongoing and to have a reasonable likelihood of being related to the study drug. Both patients had a history of diabetes mellitus.

Adverse events of special interest for treatment‐emergent infections were reported in 12 patients receiving 40 mg every week and two patients who switched to receiving 80 mg every other week, with most being mild to moderate in intensity and assessed by the investigator as having no reasonable possibility of relationship to the study drug. No safety concerns were identified in clinical laboratory tests and vital sign measurements. There were no inflammatory bowel disease AE and one (6.7%) AE of folliculitis (below the threshold used in Table 2 for reporting incidence of AE by preferred term [occurring in more than one patient]). There were no malignancies or deaths during the study.

### QoL

Mean (standard deviation [SD]) DLQI score at baseline was 5.5 (4.85) with a median (range) of 6.0 (0–15), indicating that HS impacted health‐related QoL for patients in this study. Patients treated with ADA showed improvements in several QoL assessments (Table 3). Mean (SD) change from baseline in DLQI at week 52 was –0.1 (5.8), and for those who switched to 80 mg every other week, mean change was –1.7 (4.50) at week 0x and –2.5 (3.67) at week 12x. By week 52, patients’ mean (SD) TSQM effectiveness score was 22.2 (22.02) and mean (SD) TSQM global satisfaction score was 28.6 (19.65). At week 12x for patients who switched to 80 mg every other week, mean (SD) TSQM effectiveness score was 44.4 (37.18) and mean (SD) TSQM global satisfaction score was 32.1 (24.22). Mean (SD) change from baseline in HS QoL score at week 52 was 2.5 (2.83), and for patients who switched to the 80 mg every other week dose, HS QoL score was 2.7 (1.97) at week 12x. Mean (SD) change from baseline in EQ‐5D questionnaire scores at week 52 was 0.1 (0.19) and 2.7 (27.44) in the pain dimension. At week 12x for patients who switched to 80 mg every other week, mean (SD) change from baseline in EQ‐5D score was 0.2 (0.17), and mean (SD) score in the pain dimension was 19.2 (22.45).

**Table 3:** Mean change from baseline in patient quality of life and treatment satisfaction in Japanese patients treated with adalimumab

Variable	Mean (SD), 40 mg every week (*n* = 15)	Mean (SD), 80 mg every other week (*n* = 6)
DLQI		
Week 52	–0.1 (5.80)	–
Week 0x	–	–1.7 (4.50)
Week 12x	–	–2.5 (3.67)
TSQM		
Effectiveness		
Week 52	22.2 (22.02)	–
Week 0x	–	40.7 (35.95)
Week 12x	–	44.4 (37.18)
Global satisfaction		
Week 52	28.6 (19.65)	–
Week 0x	–	34.5 (24.91)
Week 12x	–	32.1 (24.22)
HS QoL		
Week 52	2.5 (2.83)	–
Week 0x	–	3.0 (2.97)
Week 12x	–	2.7 (1.97)
EQ‐5D		
Week 52	0.1 (0.19)	–
Week 0x	–	0.1 (0.18)
Week 12x	–	0.2 (0.17)
EQ‐5D, pain dimension		
Week 52	2.7 (27.44)	–
Week 0x	–	11.7 (29.94)
Week 12x	–	19.2 (22.45)

Missing data were handled using the last observation carried forward method before week 52 and by observed cases after week 52 for all quality of life (QoL) assessments. DLQI, Dermatology Life Quality Index; EQ‐5D, EuorQol questionnaire; HS QoL, Hidradenitis Suppurativa Quality of Life questionnaire; SD, standard deviation; TSQM, Treatment Satisfaction Questionnaire–Medication.

## DISCUSSION

Results from this open‐label, phase 3 study demonstrate that weekly, long‐term treatment with ADA is effective, safe and improves patient QoL in Japanese patients with moderate to severe HS. Most patients achieved HiSCR at week 12 (primary end‐point) and the clinical response was supported by improvements at week 12 in total AN count, skin pain due to HS and modified Sartorius score (secondary end‐points), all of which were sustained through the study period. Improvements were also seen and generally maintained throughout the study in overall patient QoL, as measured by DLQI, TSQM, HS QoL and EQ‐5D assessments. The safety profile of ADA 40 mg weekly treatment and biweekly treatment with ADA 80 mg observed in this study was consistent with safety profiles in other clinical trials of ADA and no new safety concerns were identified.^2^, ^20^

The current results provide an adequate characterization of the efficacy and safety profile of ADA in the Japanese population, although limitations exist regarding sample size and the absence of a placebo or comparator treatment arm in this open‐label study. Because few studies have investigated HS epidemiology in Japanese populations,^5^, ^21^ further evaluation of the safety and efficacy of ADA treatment in this population is warranted. Compared with findings from the global PIONEER clinical trials,^2^ at week 12 a larger proportion of patients in our study achieved a clinical response with ADA, and there was a larger mean change from baseline in TSQM. Differences in the observed efficacy of ADA and QoL assessments may be attributed to differences in the study populations. For example, this study mainly enrolled men with mean body mass index of 26.5 kg/m^2^ and lesions were predominantly located in the buttock area, whereas most patients in the PIONEER trials were women with higher mean body mass index (33 kg/m^2^).^2^ Furthermore, other studies performed in Western countries have reported that HS is more common in women than men and that the most common anatomical site of lesion occurrence is in the axillary, inguinal and anogenital regions.^22^ Additional studies may identify the optimal patient profile for the use of ADA, especially for patients with pre‐existing comorbidities.

Adalimumab is the only treatment option for moderate to severe HS approved by regulatory authorities in Europe, the USA and Japan.^15^ Several other modalities are used to manage HS, including topical antibiotics for mild disease to laser/light interventions and surgery for moderate to severe disease. However, access to treatment may be limited, and there are differences in clinical treatment guidelines and surgical recommendations.^4^, ^23^ HS surgery involves excision of the affected area and often results in large skin defects and postoperative morbidity. Patients may require additional surgical procedures to close the defect including skin grafts, pedicle flaps or secondary granulation wound closures.^23^ The QoL in patients with HS is impacted in multidimensional ways, from visible lesions with odorous drainage to scarring from surgery. QoL assessment scores for patients with HS are often worse than QoL scores for patients with other dermatological conditions, malignant neoplasms, Parkinson’s disease and stroke.^4^

The results of this study demonstrate that weekly administration of ADA through 124 weeks is safe and effective for treating Japanese patients who are living with moderate to severe HS. Results from the group who switched to a less frequent dosing regimen remained similar in efficacy, safety and QoL outcomes. Similar efficacy and safety were observed in patients after their switch from ADA 40‐mg dosing every week to ADA 80‐mg dosing every other week. ADA treatment may provide even longer term benefit in clinical response, reduction in pain and improved QoL in Japanese patients with moderate to severe HS, as supported by results from another open‐label extension study (*n* = 88) where efficacy was maintained throughout 168 weeks.^24^

## CONFLICT OF INTEREST

The authors and AbbVie scientists designed the study and analyzed and interpreted the data. All authors contributed to the development of the content; all authors and AbbVie reviewed and approved the manuscript; the authors maintained control over the final content. A. M. has received research grants, consulting fees and/or speaker’s fees from AbbVie, Eli Lilly Japan, Janssen Pharmaceutical, Kyowa Hakko Kirin, Leo Pharma, Maruho, Mitsubishi‐Tanabe Pharma and Novartis. H. T., T. N. and T. M. have no disclosures to report. K. O. has received research grants from AbbVie. S. I. has served as paid speaker for and/or participated in clinical trials sponsored by companies that manufacture drugs used for the treatment of psoriasis, including AbbVie, Mitsubishi‐Tanabe Pharma, Janssen Pharmaceutical, Novartis, Eli Lilly Japan, Kyowa Hakko Kirin, Leo Pharma, Maruho, Celgene, Torii Pharmaceutical and Taiho Pharmaceutical. K. T. has served as paid speaker for and/or participated in clinical trials sponsored by companies that manufacture drugs used for the treatment of psoriasis, including AbbVie, Mitsubishi‐Tanabe Pharma, Janssen Pharmaceutical, Novartis, Eli Lilly Japan, Kyowa Hakko Kirin, Leo Pharma, Maruho, Boehringer Ingelheim, Taiho Pharmaceutical, Torii Pharmaceutical, Eisai and Celgene (Bristol‐Myers Squibb). Y. O. has received funds for research/research grants/speaker fees from Maruho, Kyowa Hakko Kirin, Eli Lilli Japan, Janssen Pharmaceutical, Celgene (Bristol‐Myers Squibb), Shiseido, Eisai, Torii Pharmaceutical and AbbVie. N. H. has received consultant fees and honoraria for serving as a speaker from AbbVie and Maruho. T. T. has received research funds from Maruho, and honoraria for speaker, consultancy and advisory board membership from AbbVie, Boehringer Ingelheim, Celgene, Janssen, Kyowa Hakko Kirin, Leo Pharma, Lilly, Mitsubishi Tanabe Pharma, Novartis and Sanofi. Y. Z., S. K. and K. T. are full‐time employees of AbbVie and may own stock/options. N. M. was an AbbVie employee at the time of the study.
